# Data sharing and reuse in clinical research: Are we there yet? A cross-sectional study on progress, challenges and opportunities in LMICs

**DOI:** 10.1371/journal.pgph.0003392

**Published:** 2024-11-20

**Authors:** Naomi Waithira, Mavuto Mukaka, Evelyne Kestelyn, Keitcheya Chotthanawathit, Dung Nguyen Thi Phuong, Hoa Nguyen Thanh, Anne Osterrieder, Trudie Lang, Phaik Yeong Cheah

**Affiliations:** 1 Nuffield Department of Medicine, Centre for Tropical Medicine and Global Health, University of Oxford, Oxford, United Kingdom; 2 Mahidol-Oxford Tropical Medicine Research Unit, Bangkok, Thailand; 3 Oxford University Clinical Research Unit, Ho Chi Minh City, Vietnam; 4 Health Data Research, London, United Kingdom; University of Washington Bothell, UNITED STATES OF AMERICA

## Abstract

Data sharing holds promise to accelerate innovative discoveries through artificial intelligence (AI) and traditional analytics. However, it remains unclear whether these prospects translate into tangible benefits in improving health care and scientific progress. In this cross-sectional study, we investigate current data reuse practices and explore ways to enhance the use of existing data in clinical research, focusing on low- and middle-income countries. 643 clinical researchers and data professionals participated in the study. 55.5% analysed clinical trial data. 75.3% of data users analysed data from observational studies obtained mainly through personal requests or downloads from publicly available sources. Data was mainly used to influence the design of new studies or in pooled and individual patient-level data meta-analyses. Key benefits realised were career progression and academic qualification, with more gains reported by users affiliated with high-income and upper-middle-income countries (p = 0.046, chi = 8.0). Scientific progress through publications and collaborations was associated with gender (p = 0.012, chi = 10.9), with males more likely to contribute. Benefits to the public although minimal, were associated with career seniority (p = 0.001, chi = 18.8), with works by senior researchers being more likely to influence health policy or treatment guidelines. Although 54% of the respondents accessed at least 3 datasets in the past 5 years, 79.4% of data users encountered difficulty finding relevant data for planned analyses. Researchers affiliated with low and middle income institutions reported more difficulty interpreting data (p = 0.012, chi = 25.7), while challenges with language were regionally influenced (p = 0.000, chi = 51.3) and more commonly reported by researchers in Latin America and South and East Asia institutions. While the utilisation of shared data is lower than expected, focused efforts to enrich existing data with extensive metadata using standard terminologies can enhance data findability. Investment in training programmes, building professional networks, and mentorship in data science may improve the quality of data generated and increase researchers’ ability to use existing datasets.

## Introduction

### The promise and potential of data sharing

Data sharing is crucial for scientific progress, allowing researchers to build upon the work of others and make novel inferences from existing data. In clinical research, reuse of shared research data is instrumental not only in evidence synthesis but also for operational purposes such as streamlining clinical research design and influencing participant recruitment strategies [1, 2]. Ultimately, data sharing and reuse aims to improve health outcomes through improved preventative, curative and rehabilitative services.

Recent studies have documented the positive impact of data reuse in healthcare. A meta-analysis combining data on 34,178 pregnancies from 12 cohorts generated evidence that resulted in a change in the World Health Organization’s guidelines on treating malaria in pregnant women [3]. In a large-scale analysis of shared genetic data, researchers identified new risk variants associated with Alzheimer’s [4]. Similarly, the reuse of electronic health records (EHRs) has facilitated the development of machine learning algorithms for predicting patient outcomes [5].

With the rapid advancements in the development of Artificial intelligence (AI)-based applications since 2022, there is potential to transform how research is conducted and how medical services are delivered. Large Language Models like GPT-4 and Bard have demonstrated capabilities to enhance academic writing, generate new research ideas, and develop software code [6, 7]. Multimodal AI, such as Google’s Gemini, can integrate diverse information such as medical images, textual patient records and genetic data to produce richer, more accurate insights than LLMs [8]

However, numerous studies have highlighted an underwhelming effectiveness of AI-based applications in healthcare such as high error rates in addressing oncology queries and misleading risk assessments for patients with chest pain [9, 10]. Additionally, ethical and legal concerns regarding data privacy and the perpetuation of racial and gender biases have been raised, prompting a need for appropriate safeguards and regulation on the use of AI in health [11, 12]. It is critical that AI models are trained using representative datasets and are evaluated in real-world scenarios.

The accuracy of both AI and traditional computation algorithms depends on using well-described and reliable data.

### Efforts to promote data reuse in clinical research

Acknowledging this need, interventions have been established to promote a culture of data sharing and reuse. These include data sharing policies, mandates, and incentives from the research communities, funders and publishers. Funding bodies such as the National Institutes of Health (NIH), the Bill and Melinda Gates Foundation, and the Wellcome Trust have mandated sharing data generated by research projects they fund [13]. Scientific journals such as the Nature portfolio, PLOS and the ‘International Committee of Medical Journal Editors’ journals now recommend or require that scholarly publications include data accessibility statements [14]. Research institutions are also acting by establishing internal data-sharing policies and setting up repositories to store and share data [15].

Introduced in 2016, the FAIR Guiding principles (Findability, Accessibility, Interoperability, and Reusability) have significantly increased the standardisation and accessibility of data [16]. This is particularly crucial in medical research, where data is obtained from heterogeneous sources in non-standard formats, and issues regarding the confidentiality of personal data are prominent. Strides have been made in defining workflows and guiding frameworks for implementing FAIR principles in health data in industry and academia [17–19]. In response to privacy concerns, initiatives to tackle ethical issues and legal frameworks for data protection, such as the European Union General Data Protection Regulation (GDPR), have been established. In addition, data science competitions and datathons are increasingly organised to cultivate a culture of data use [20, 21]. These forums bring together multi-disciplinary teams of data experts and clinicians. Data experts use their computational skills to generate new findings from data, while clinicians contribute contextual and medical interpretation of the findings.

### Amid progress, challenges persist

Despite these efforts, clinical research appears to lag behind other scientific fields in data reuse [22, 23]. A review of data used in openly accessible clinical trials repositories that included Yale University Open Data Access Project (YODA), ClinicalStudyDataRequest (CSDR), and the Supporting Open Access for Researchers (SOAR) found that only 15.7% of over 3,000 datasets in the repositories were requested [24]. Other literature suggests that the proportion of requests resulting to a publication was very low (CSDR:18.6%, YODA:16.8%, VIVLI:6.5%) [2]. These studies attribute the low rates of scientific output to data quality, inappropriate study design, and lack of sufficient metadata. Additionally, limited access to analytical tools and concerns about potential exploitation diminish the enthusiasm of some researchers to share and utilise data [2, 24–26].

A substantial amount of scholarly work has been dedicated to discussing the concerns and merits of the sharing of data by those who have collected the data. However, far too little attention has been paid to how shared data is used and the consequent impact in achieving data sharing objectives in clinical research. Current literature is predominantly based on high-income countries, resulting in a substantial knowledge gap concerning practices and the impact of data use in low- and middle-income countries (LMICs). This study sought to address the knowledge gap by examining data reuse practices in clinical research, focusing on the LMIC context. We identified key factors that hinder data utilisation and solicited practical solutions from the perspective of researchers and data experts. We anticipate that our findings will contribute to further research and development of evidence-based solutions for promoting effective, ethical, and equitable data reuse.

## Methods

We conducted mixed-method research that included a cross-sectional survey and a qualitative study. This paper presents the results of the cross-sectional study conducted as a self-administered, web-based survey.

### Ethics statement

The protocol was approved by the Oxford Tropical Research Ethics Committee (reference number: 568–20) and is published online [27]. Written informed consent was obtained before respondents took the survey. The survey was anonymous and did not capture personal identifying information. A statement on data storage and protection was provided in the information sheet for respondents’ review and consent before participation.

### Data collection

The survey instrument contained 16 questions, grouped into four main sections: Respondents’ demographic characteristics, Nature of data use, Challenges with data use, and Solutions to enhance data use. Questions were presented in multiple-choice, Likert scale, and open-ended formats. Conditional logic was applied to show questions based on supplied responses; for instance, participants who did not generate outputs from secondary analyses were not asked to describe types of outputs. All displayed questions were mandatory. Participants were allowed to save incomplete responses for later completion, but this was permissible only within 24 hours of initiating the survey.

The questionnaire was developed in English and was piloted with 16 participants. Ambiguities in wording were corrected before the survey was released. To achieve sociodemographic diversity, professional translators translated the survey into Spanish, French, Portuguese, and Vietnamese and validated by an independent translator. Native speakers similar to the target population verified the translated surveys for psychometric equivalence. The questionnaires were developed into web-based surveys using the JISC tool managed by the University of Oxford. Between 05^th^ April 2021 and 30^th^ Nov 2022, the survey was distributed in seminars, medical conferences professional networks for researchers and data users working in LMIC, and through social media channels such as Twitter, Facebook, and LinkedIn. Participants were encouraged to share the survey with their colleagues and professional networks.

### Statistical considerations

The sample size was determined using a non-probability sampling approach due to the inherent difficulties in achieving a genuinely random sample in a global and online context. Using the precision method for sample size calculations, a minimum of 200 participants would suffice to describe the study population and estimate the prevalence of a response, assuming a 50% prevalence rate, with a confidence level of 95% and a margin of error of 7%. In the absence of a well-known prevalence of an outcome, an assumption of 50% prevalence is used as a rule of thumb in sample size calculations. This assumption leads to the largest minimum sample size for a given margin of error and a confidence level. Our sample size estimate implies that a sample size higher than 200 participants would increase the precision of prevalence estimates. Therefore, no upper limit was defined for the total number of respondents to be enrolled.

The following formula was used to calculate the sample size: [28]

$$n=\frac{p(1-p)}{{\left(E/1.96\right)}^{2}}$$

Where p is the prevalence of outcome expressed as a proportion, i.e., 0.5 in our case, E is the margin of error, 0.07, and 1.96 is the standard normal z-value corresponding to the 95% confidence interval. The sample size, power calculations and analyses were made in Stata 18. Descriptive statistics were stratified by self-reported data use history and evaluated using chi-squared tests at a 5% significance level (i.e., alpha = 0.05).

## Results

### Participant characteristics

A total of 995 participants started the survey, and 643 of them submitted complete responses. Duplicate entries were screened by comparing demographic characteristics. No duplicates were identified, and all complete responses were included in the analysis.

The final sample, comprising 643 participants, represented diverse academic disciplines that included Infectious Diseases, Global /Public Health, Clinical Laboratory Sciences (microbiology, pharmacology, omics, molecular biology), Epidemiology, and others (paediatrics, gastroenterology, intensive care medicine). The majority of respondents were early to mid-career researchers affiliated with academic and public institutions. 54.1% of the participants reported a history of using data shared by other researchers. Table 1 summarises demographic characteristics stratified by the respondents’ data use history.

**Table 1:** Demographic characteristics stratified by history of data use.

	Data user (N = 348)	Non-user (N = 295)
**Occupation: n (%)**		
Clinical researcher	205 (58.9)	206 (69.8)
Informaticians (data scientists, statisticians, bioinformaticians)	21 (6.0)	0 (0.0)
Research support professional	50 (14.4)	59 (20.0)
Other	72 (20.7)	30 (10.2)
**Career-level: n (%)**		
Senior researcher	40 (13.5)	34 (13.3)
Mid-career researcher	52 (17.6)	45 (17.6)
Early career researcher	78 (26.4)	82 (32.0)
Graduate student	31 (10.5)	40 (15.6)
Research support professional	95 (32.1)	55 (21.5)
**Gender: n (%)**		
Male	157 (45.98)	116 (39.32)
Female	185 (53.45)	171 (58.64)
Other	1 (0.29)	0 (0)
Prefer not to say	1 (0.29)	6 (2.03)
**Age group: n (%)**		
18–24	12 (3.45)	11 (3.73)
25–34	122 (35.06)	104 (35.25)
35–44	135 (38.79)	120 (40.68)
45–54	50 (14.37)	42 (14.24)
55–64	23 (6.61)	12 (4.07)
65–74	6 (1.72)	5 (1.69)
Prefer not to say	0 (0)	1 (0.34)
**Sector: n (%)**		
University or Academic Research Organisation	194 (56.4)	149 (50.9)
Government or public institution	63 (18.3)	46 (15.7)
Non-Governmental or Faith-Based Organisation	24 (7.0)	36 (12.3)
Commercial organisation (e.g. pharmaceutical company)	15 (4.4)	11 (3.8)
Regulator	10 (2.9)	15 (5.1)
Research Funder	4 (1.2)	3 (1.0)
Other	34 (9.9)	32 (10.9)

*Research support staff include project managers, laboratory staff, research coordinators

**Fig 1:**
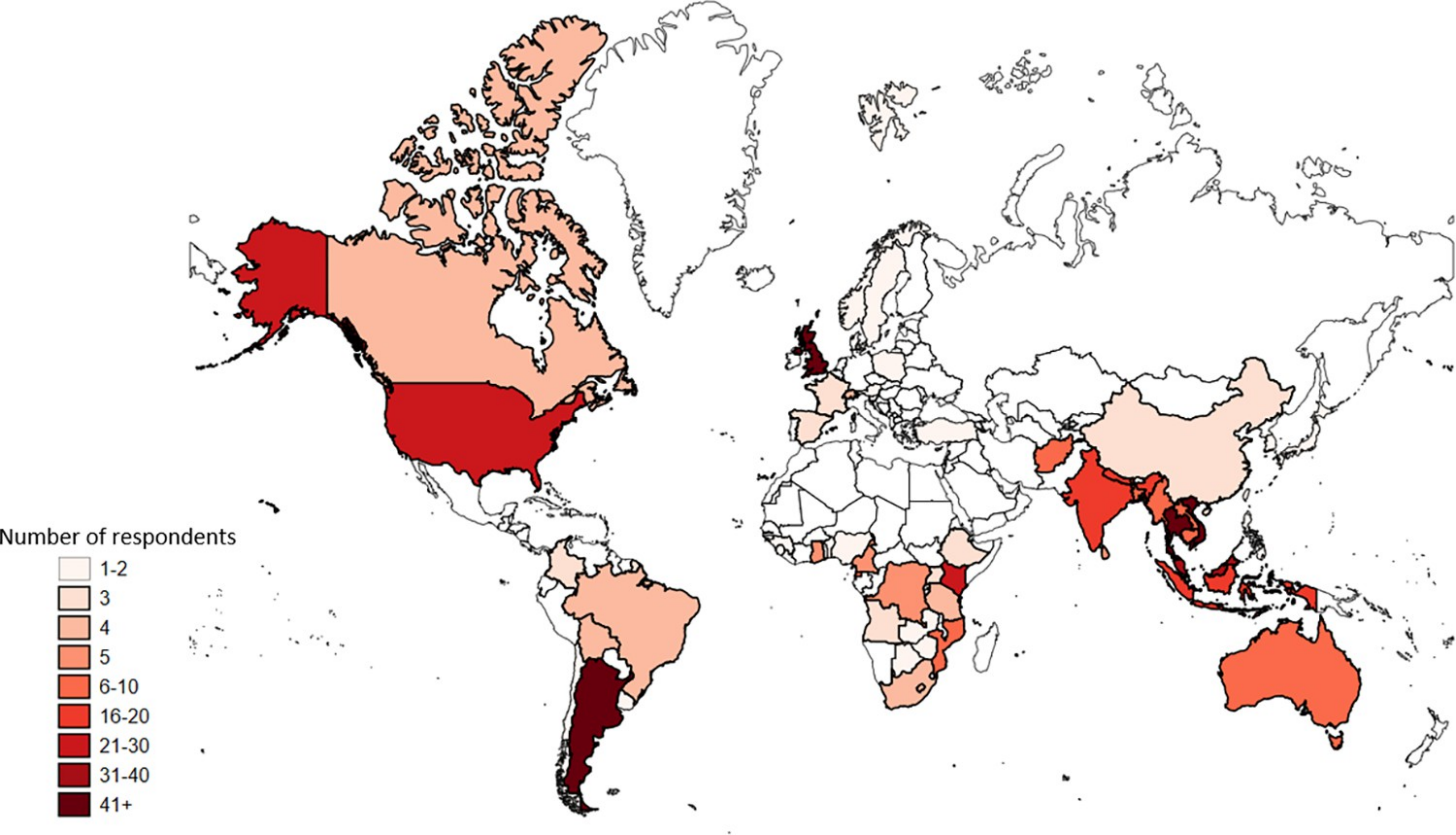
Geographical location of respondent’s employers.

We captured the geographical location of participants’ employers and used the World Bank Region Classification to categorise the 68 countries based on their income levels [29]. Most respondents (290, 45.1%) were employed by institutions in Low and Middle-Income Countries, as detailed in Table 2. Fig 1 shows the geographic location of the participants’ employers.

**Table 2:** Distribution of the employer’s country by income.

	Data users (N = 348)	Non-users (N = 295)
High income, n (%)	70 (20.3)	47 (16.0)
Upper middle income, n (%)	139 (39.2)	97 (32.4)
Lower middle income, n (%)	130 (37.8)	134 (45.7)
Low income, n (%)	9 (2.6)	17 (5.8)

### Nature of data use

Our study indicates that nearly half of the data users (44.3%) obtained data from public repositories, 39.5% requested data directly from data custodians or collectors, 14.5% accessed data via a data access committee, and 1.8% indicated using other avenues. These datasets primarily contributed to shaping the design of new studies (39.1%) or were utilised in pooled or meta-analyses (29.5%), as reflected in Table 3. Data use for algorithmic purposes was low: only 9.6% of the respondents reported using data to develop or validate models or AI algorithms.

**Table 3:** Purpose of data request.

Purpose	N (%)*
Study design	251(39.1)
Pooled or meta-analyses	190 (29.5)
Educational or exploratory	91 (14.2)
Model or Algorithm development	62 (9.6)
Validation or reproduction	57 (8.9)
Policy or regulatory	50 (7.8)
Other	14 (2.2)

*cumulative proportions exceed 100% as respondents may have used data for multiple purposes

**Fig 2:**
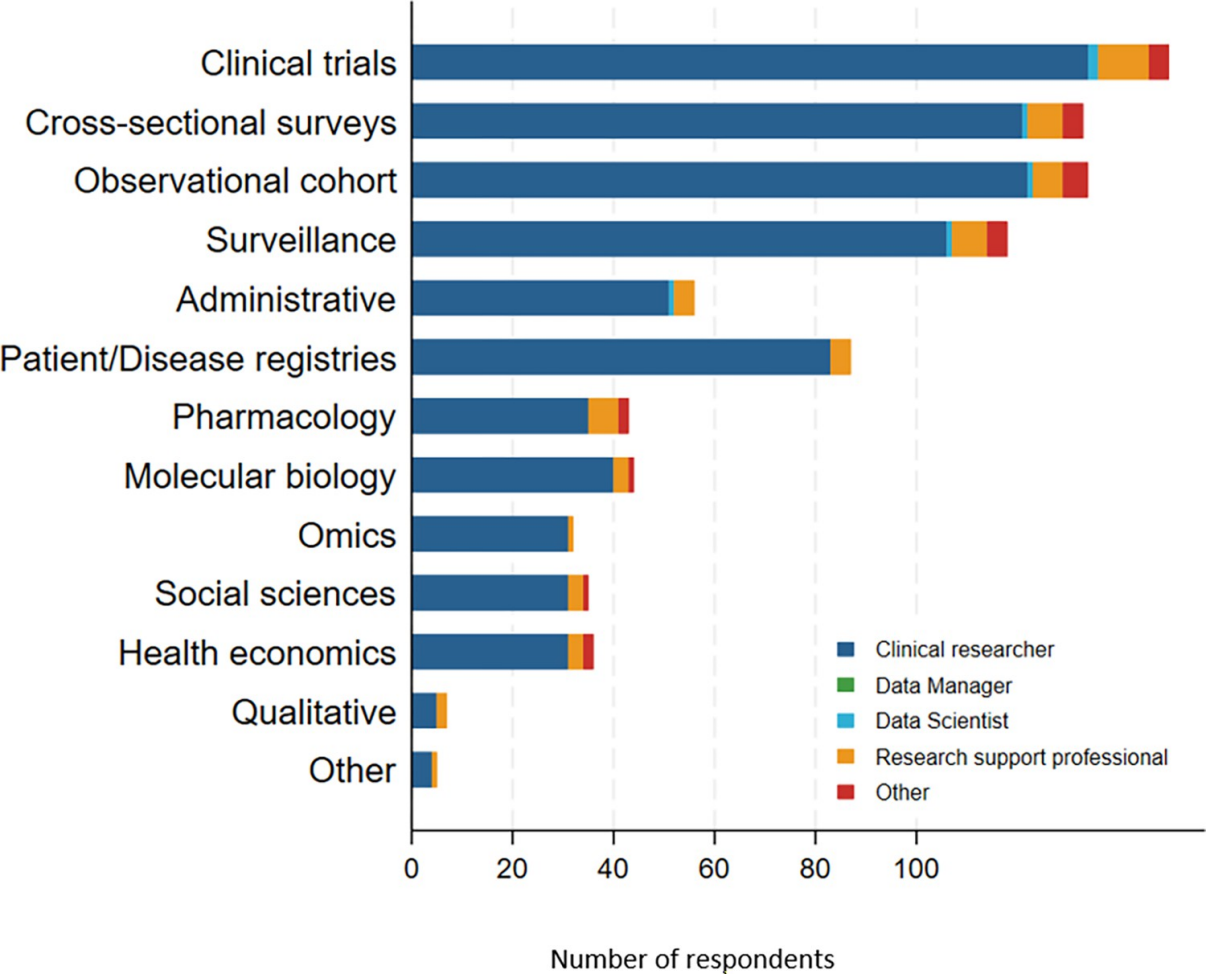
Data types and occupation.

The majority of users (262, 75.3%) utilised data from observational studies, such as cross-sectional surveys, cohort studies, and surveillance studies. Clinical trial datasets were utilised by 193 data users, representing 55.5% of the cohort. Fig 2 provides a breakdown of the types of data used stratified by occupation.

We examined the reasons why non-users did not use shared data. 52.9% mentioned that they did not need to use others’ data, 22.4% struggled to find specific data and 12.5% could find the data but were unable to access it. 3.7% had difficulty using the data, and 8.5% cited other reasons, such as hesitance to request data from other researchers as they were new in the field or expecting to need others’ data in the future.

### Outputs

A predominant number of respondents produced 1 to 4 outputs from their analyses in the form of publications, presentation materials for conferences and meetings, and academic theses and reports. It is notable that most outputs comprised grey literature: 62.9% of data users generated material used in presentations such as conferences and seminars. In comparison, 36.5% used data in writing academic theses. Peer-reviewed publications were reported by 58.1% of the data users. 5.8% of data users conducted secondary analyses and generated no outputs, as illustrated in Table 4.

**Table 4:** Outputs generated in the last 5 years.

Output	N (%)
Presentations	219, 62.9%
Publications	202, 58.1%
Thesis	127, 36.5%
Report	107, 30.8%
Social Media	32, 9.2%
Book chapter	21, 6.0%
None	20, 5.8%
Other	17, 4.9%
Mathematical models	16, 4.6%
AI	11, 3.2%
Software	8, 2.3%

*cumulative proportions exceed 100% as respondents may have generated multiple outputs

Further analysis revealed an association between the type of outputs generated and the researchers’ occupation, career level, and employer.

Senior and mid-career researchers produced more publications and book chapters (p = 0.028, chi = 9.1), a trend similarly observed among male researchers compared to their female counterparts (p = 0.002, chi = 14.8). Algorithmic outputs, such as mathematical models and software code, were related to the occupation (p<0.001, chi = 44), with modellers, programmers, and data managers contributing more than clinical researchers. Additionally, more publications were generated by respondents affiliated with institutions in high-income countries and upper-middle-income countries (p = 0.028, chi = 9.1).

### Benefits

Our analyses revealed a statistically significant relationship between personal benefits from secondary data use and the geographical location of the respondent’s employer. Users affiliated with high-income and upper-middle-income countries reported more personal gains through career advancement, financial gains, and attaining higher education (p = 0.046, chi = 8.0).

Scientific progress through the generation of new knowledge, as well as validation or correction of established findings, were associated with gender (p = 0.012, chi- = 10.9). Male users had a higher likelihood of scientific contribution.

An association was observed between benefits to the general public, such as updates to health policies and improved access to drugs and medical devices, and the career level of researchers. Senior researchers demonstrated a higher likelihood of influencing strategic policy decisions and fronting the registration of new medicines and medical devices (p = 0.001, chi = 18.8).

### Barriers and enablers of data use

#### Barriers

Data users ranked challenges encountered in accessing and utilising data and their effect on planned analyses using a Likert scale (1 to 5). A rating of 1 indicated that the issues were not encountered, 2 showed no impact, 3 denoted low impact, 4 reflected moderate impact, and 5 signified high impact. The challenges identified were classified into four overarching themes aligned with the FAIR principles: Findability, Accessibility, Interoperability, and Reusability, as detailed in Table 5. In the following paragraphs, we outline the proportion of respondents who experienced a challenge, regardless of its impact on planned analyses.

**Table 5:** Classification of challenges based on the FAIR principles.

Category	Challenge	Not experienced N(%)	No impact (%)	Low impact N(%)	Moderate impact N(%)	High impact N(%)
Findability	Difficulty finding relevant data	71(20.5)	25(7.2)	84(24.2)	119(34.2)	48(13.8)
	Data not available at the time of publication of research findings	112(32.2)	42(12.1)	81(23.3)	78(22.5)	34(9.8)
	Data no longer exists in the repository	156(44.9)	46(13.3)	66(19)	54(15.6)	25(7.2)
Accessibility	Unclear process for accessing the data	71(20.5)	52(15)	89(25.6)	86(24.8)	49(14.1)
	Excessive bureaucracy	84(24.2)	44(12.7)	91(26.2)	90(25.9)	38(11)
	Slow or no response from the data provider	106(30.5)	43(12.4)	78(22.5)	72(20.7)	48(13.8)
	Denied access to data	151(43.4)	41(11.8)	60(17.3)	43(12.4)	52(15)
	The cost of data was prohibitive	168(48.3)	39(11.3)	47(13.6)	52(15)	41(11.8)
Interoperability	Limited or no metadata (data dictionary, protocol, statistical analysis plan)	120(34.5)	39(11.3)	92(26.5)	57(16.4)	39(11.3)
	Difficulty understanding the data	81(23.3)	41(11.8)	107(30.8)	79(22.8)	39(11.3)
	Data was in a different language	155(44.6)	54(15.6)	63(18.2)	53(15.3)	22(6.4)
	Unusable data format or structure	103(29.6)	40(11.5)	97(27.9)	74(21.3)	33(9.5)
Reusability	Data was provided with investigator-imposed restrictions	129(37.1)	42(12.1)	70(20.2)	67(19.3)	39(11.3)
	Ethical, legal, or privacy restrictions	113(32.5)	52(15)	68(19.6)	69(19.9)	45(13)
	Data variables needed were not collected in the dataset	85(24.5)	34(9.8)	79(22.8)	95(27.3)	54(15.6)
	Errors or inconsistencies in data	85(24.5)	34(9.8)	104(29.9)	72(20.7)	52(15)
	Incompleteness of data (many values were missing)	74(21.3)	30(8.7)	95(27.3)	82(23.6)	66(19)
	Inappropriate study design (such as inappropriate outcome measures)	119(34.2)	46(13.3)	101(29.1)	60(17.3)	20(5.8)
	Insufficient data (e.g. sample size too small)	98(28.2)	37(10.7)	86(24.8)	93(26.8)	33(9.5)
	Lack of resources to use data	135(38.8)	46(13.3)	83(23.9)	54(15.6)	29(8.4)

Our results indicate that challenges with ‘Findability’ mainly related to difficulty tracing data for planned analyses (79.4%) and unavailability of data at publication (67.7%). These challenges were reported to have a mainly moderate impact on planned analyses.

Accessibility challenges included unclear processes for accessing data (79.4%), excessive bureaucracy (75.5%), and slow or no response by data providers (69.4%). These challenges had a low to moderate effect on planned analyses for most respondents.

Interoperability challenges were mainly characterised by difficulty understanding the data, unusable data formats, and a lack of metadata. They had a low impact on planned analysis for most respondents.

Reusability challenges are related mainly to data quality, characterised by missing data, errors, and inadequate sample sizes. These challenges had a low to moderate effect on planned analyses for most of the respondents.

Our analysis shows that 79.4% of data users experienced the challenge of finding required data. This challenge also had the most significant negative impact on planned projects, with a moderate effect on 34.2% and a high impact on 13.8% of planned analyses. These challenges were observed across the respondent groups, with no significant relationship with the dependent variables.

Challenges related to unusable data formats and difficulty understanding the data were more prevalent among researchers with LMIC affiliation (p = 0.012, chi = 25.7). Problems with data in a different language were regionally influenced (p<0.001, chi = 51.3) and were reported by researchers in Latin America, South Asia and East Asia institutions. Respondents from South Asia experienced a higher impact on planned analyses than those from other regions.

Lastly, lack of access to analytical resources was not a significant concern for respondents from High-Income Countries (HIC). Still, those from Upper-Middle-Income Countries (UIC) and LMIC respondents reported a moderate to high impact, although the proportions remained low (11% and 16% respectively).

#### Enablers

All respondents(n = 643) systematically ranked interventions that would increase data use in clinical research using a Likert scale (1 to 5, where 1 is least important and 5 is most important); Table 6 presents summary statistics of the solutions. Participants at all career stages ranked access to information about the location of clinical research datasets highest. Additionally, they deemed guidance on research etiquette, including authorship, attribution, and responsibilities, as a key enabler. Research support professionals and early career researchers ranked training and access to analytical tools highest.

**Table 6:** Summary statistics of enablers ranked by importance.

	Mean (95% Confidence Interval)
Repositories: where to find relevant data	4.4 (4.4 to 4.5)
Authorship, attribution, responsibilities	4 (3.9 to 4.1)
Analysis methods and tools	4 (3.9 to 4.1)
Data licensing	3.8 (3.7 to 3.9)
Financial assistance	3.6 (3.5 to 3.7)
Legal assistance	3.6 (3.5 to 3.7)

## Discussion

In this study, we investigate data reuse practices in clinical research and highlight benefits, barriers, and enablers from the perspective of data users. We aim to contribute insights to the ongoing discussion on increasing the scientific and social impact of data sharing in health.

### Nature of data use

Our study reveals that the primary motivation for secondary data use was to generate new insights and to influence the design of new research projects. Despite the widely recognised significance of reproducibility in scientific research [30], our findings indicate that reproducibility is not a key motivator for secondary analyses. This may be attributed to the academic reward system prioritising innovation over reproducibility [31]. The primary output from secondary analyses was grey literature, such as conference presentations, reports, and theses. Although publications serve as the primary metric for assessing research productivity, the impact of grey literature should not be underestimated, particularly in tracking research output and its subsequent impact.

As expected, the use of data to develop or validate algorithms was associated with computational professions such as bioinformaticians, statisticians, and modellers. Our findings indicate a low level of data utilization for AI-related applications, which may be attributed to demographic factors or a general tendency to underutilize data for training AI models. Further investigation is warranted to analyze patterns of reuse of health research data in the development and validation of AI applications.

We note that data from observational studies was used at higher rates compared with that from Randomized Controlled Trials (RCT). Considering that RCTs are perceived as the gold standard for evaluating health interventions [32], questions arise on what specific barriers impede the use of RCT data. Previous work has suggested that ethical and legal challenges are significant contributors, proposing broad consenting models and regulated data access through Data Access Committees (DACs) as solutions [33–35]. However, our study shows that data is often obtained directly from data custodians or downloaded from public sources. A plausible explanation is due to the delays associated with DACs, such as the time taken to prepare data requests, negotiate data-sharing agreements, and engage in administrative correspondence. Conversely, institutions perceive DACs as a mechanism to mitigate the risks associated with ethical and legal issues in data sharing [36].

### Impact

While impact may take a long time to manifest, there are discernible intermediate benefits. We describe these benefits in three dimensions: to the individual, scientific research, and the general public’s health.

Benefits to the researcher include career advancement, remuneration, and academic qualifications. A correlation was observed between individual benefit and the economic status of the data user’s employer. Users attached to institutions in high-income countries experienced more personal benefits than counterparts from other regions. Previous work suggests that data sharing and reuse are driven by factors other than altruism, [26] and while we cannot ascertain this claim from our results, we note that institutions in high-income countries prioritise secondary data analysis by offering extensive training programs and providing access to advanced computational resources like high-performance computing systems and analytical tools. Additionally, broad professional networks serve as a valuable asset for mentorship, enabling inexperienced researchers to acquire insights and experiences from their more seasoned counterparts [37]. Implementing similar strategies in low- and middle-income country settings can foster growth and progression for researchers and a more equitable landscape.

We consider scientific impact from the perspective of creating new knowledge, developing innovative research ideas, and confirming or correcting known facts. Scientific contribution, primarily through publications, showed a notable correlation with gender. Despite having a slightly higher number of female respondents in the study, male respondents reported more outputs. This corresponds with previous literature highlighting gender disparities in scientific authorship and leadership [38, 39]. There are efforts targeted at fixing the ‘*leaky pipeline’* in biomedical disciplines, where there is a disproportionate reduction in the number of women progressing through the career stages [40]. As expected, the contribution of algorithmic outputs, such as mathematical models, software validation, and code, was significantly influenced by occupation. Although a few clinical researchers generated software code, most of the contribution was from bioinformaticians, mathematical modellers, and statisticians.

Benefits to the general public are characterised by increased access to drugs and medical devices and influence on health policies. These were reported by a low proportion of the respondents (1.4% for new drug registration and 13.5% for input into health policies or guidelines), the majority of whom were seasoned researchers in senior and mid-career positions. Although the figures suggest a potential gap in translating research findings into tangible public health outcomes, it is essential to note that achieving a substantial impact on health is gradual and takes time. To effectively monitor the long-term impact of data sharing on health outcomes, it is essential to consistently track metrics relating to patient health, disease management, and healthcare accessibility over time, along with incorporating qualitative feedback from stakeholders. Data citation metrics are a valuable tool for tracking datasets and evidence that support policy decisions. They can provide an objective, albeit partial, measure of the impact of data sharing and reuse on health outcomes.

### Barriers and solutions

Based on the FAIR principles, we outline the barriers reported by data users, the solutions proposed by researchers, and our reflections.

### Findability

Our study reveals a staggering statistic: 79.4% of data users encountered difficulties in locating data for their planned analyses. We attribute challenges with data discoverability to several factors. Firstly, clinical research data is stored across different platforms, including institutional databases, data repositories, and storage devices such as hard drives. While journals encourage or mandate statements on data availability in publications, these statements are often vague or missing altogether [41]. Search engines, such as Google Dataset Search, can help discover datasets. They, however, rely on the premise that the datasets are well-annotated with rich metadata that conform to common standards like schema.org. A recent study suggests that only 18% of biological repositories utilise a common data standard for presenting metadata [42]. As a result, search engines are unable to retrieve accurate results leading to obscurity of certain datasets. Efforts have been made by data journals, including Scientific Data by Nature, Open Health Data by Ubiquity Press, and Data in Brief by Elsevier, to publish data articles. Data articles provide detailed descriptions of datasets, including methodology, context, and background information on data collection, processing, and analysis techniques. Data articles are identified using a persistent identifier, DOI, that facilitates proper citation and attribution. However, researchers’ awareness of their existence and utility is still low. Additionally, there is potential for publication bias as well-curated or well-known datasets are most likely to be published. Scholarly databases like PubMed and Scopus often incorrectly index and label data articles as regular research papers, hindering their visibility [43]. Low data discoverability is further compounded when data are held in silos and behind firewalls with restricted access often imposed for privacy, security, or licensing reasons. Variations in the language and terminology used to describe data can cause users to overlook valuable datasets, even when they are openly available.

Tackling the challenges of data discoverability requires a multipronged approach. Assigning datasets persistent identifiers such as Digital Object Identifiers (DOIs) provides them with a unique and permanent key, through which they can be discovered and cited. DOIs can be assigned by publishing datasets as articles or uploading data to well-recognized repositories. Next, integrating dataset Digital Object Identifiers (DOIs) into publications and research outputs creates a transparent link between scholarly work and the underlying datasets, ensuring proper attribution. Lastly, describing the data with appropriate and standardised tags enhances searchability for both human users and algorithms. In instances where access to data needs to remain restricted, keeping the metadata (DOIs, tags, and descriptive documentation) public allows potential users to discover the data but safeguards privacy by mandating appropriate authorisation before accessing data.

### Accessibility

The challenges related to data access are primarily attributed to bureaucratic processes, which are further complicated b ystrict data privacy regulations. Additionally, data providers’ lack of interest in contributing data can lead to significant delays.

We advocate that primary data collectors should release data following a defined embargo period, if necessary. Sharing data along with comprehensive metadata not only facilitates further analyses by fellow researchers but also promotes a sense of altruism and supports the overarching objective of advancing public health. Additionally, obtaining consent from research participants for the future use of their data, along with the implementation of robust safeguards such as deidentification and data anonymisation, can address the challenges associated with privacy and ethics.

**Interoperability** challenges stem from diversity in how data are created, presented, and described. Variability in data causes challenges when comparing or combining data from different sources. We anticipate that low volumes of available data may, in part, drive the low outputs of AI-based models by our respondents. Reliability and generalizability of mathematical and AI models are dependent on the quality and representativeness of training data. Where data are heterogeneous and scarce, as in the case of LMIC research, the algorithms may arrive at biased or incorrect conclusions.

There are numerous initiatives to standardise data and metadata definitions by promoting consistent data collection practices. The NIH Common Data Element (CDE) Repository (https://cde.nlm.nih.gov/home) is a resource developed by the National Institutes of Health to standardise data elements in clinical research, genetics, imaging, behavioural sciences, biospecimens, and environmental exposures. The CEDAR platform (https://cedar.metadatacenter.org) facilitates the creation of standardised, machine-readable metadata based on established standards and ontologies, while the HL7 (Health Level Seven) and FHIR (Fast Healthcare Interoperability Resources) standards in healthcare information technology aid the exchange and integration of health information across systems. Implementing these standards has the potential to increase the volume of data available and reduce curation efforts. However, compliance and adoption of these standards across organisations requires skilled staff and a cultural change.

**Reusability** challenges were reported in three main dimensions: concerns over data quality, the absence of standardised outcomes measures, and unclear context on the datasets.

Data quality issues such as errors and missing values can be dealt with through statistical methods such as imputation and sensitivity analyses. However, the validity of the results may be compromised if errors are widespread or systematic. A more effective solution is to minimize errors and missingness through appropriate data management practices during data collection.

Non-standardized methods for defining outcomes can pose challenges in synthesizing research findings and comparing study results particularly in pooled or meta-analyses. Core Outcome Sets(COS) attempt to address heterogeneity in outcome measurement. A COS is *"an agreed standardized set of outcomes that should be measured and reported*, *as a minimum*, *in all clinical trials in specific areas of health or health care*.*"* [44]. COS such as (https://www.comet-initiative.org) are developed through consensus among clinicians and patients and aim to address problems with heterogeneity and to improve cross-study comparability.

Interpreting data without sufficient contextual information increases the likelihood of incorrectly rejecting a true null hypothesis or basing conclusions on spurious relationships. Sharing data research protocols, data dictionaries and, where available, a statistical analysis plan alongside datasets will aid future users in understanding the semantic context, the units of measurement, and any transformations or calculations applied to data.

## Limitations

While this study offers insights into data reuse practices in the clinical research community, it is essential to acknowledge its limitations. As the survey was targeted at researchers working in LMIC or with LMIC data, there is potential for sampling and response bias, which could affect the external validity and generalizability of the findings. The survey’s web-based and anonymous nature prevented the establishment of a sampling frame. Reliable population parameters that could be used to weight the results to account for sampling differences were challenging to obtain. As such, unweighted results have been summarised and reported. As a cross-sectional study, our data does not establish temporality and causation in the rapidly evolving landscape of data sciences due to advances in AI and the effect of data-related regulations.

To address limitations due to bias, we conducted in-depth interviews alongside the survey allowing purposeful inclusion of diverse perspectives. The interviews offer a richer understanding of participants’ experiences and, when triangulated with survey results, may enhance external validity and interpretation of the survey results. We anticipate that the findings from this study will serve as a foundation for future research to investigate and validate the observed associations.

## Recommendations and further work

Our study shows substantial progress yet to be made in the use of shared data within clinical research. Challenges in data discoverability and access contribute significantly to this limited use. In light of the findings presented in this study, the following recommendations are proposed to guide future research and inform practical implications in promoting use of existing data in clinical research:

1. Assigning persistent identifiers, such as DOIs, to research outputs, including datasets, software code, and grey literature, to facilitate discoverability, citation, and provenance.
2. Establishing a minimal set of metadata fields to be captured for research outputs and then annotating existing datasets with comprehensive metadata to enhance data discovery by humans and search engines.
3. Investing in data science training, mentorship and increased access to analytical infrastructure, particularly for LMIC researchers, to improve the quality and integrity of data generated and equip researchers with the skills to use existing data.

## Supporting information

S1 Checklist Inclusivity in global research. (DOCX)
